# Long-term fluoxetine treatment induces input-specific LTP and LTD impairment and structural plasticity in the CA1 hippocampal subfield

**DOI:** 10.3389/fncel.2013.00066

**Published:** 2013-05-09

**Authors:** Francisco J. Rubio, Estíbaliz Ampuero, Rodrigo Sandoval, Jorge Toledo, Floria Pancetti, Ursula Wyneken

**Affiliations:** ^1^Laboratorio de Neurociencias, Centro de Investigaciones Biológicas, Universidad de los AndesSantiago, Chile; ^2^Laboratorio de Neurotoxicologia Ambiental, Universidad Católica del NorteCoquimbo, Chile

**Keywords:** antidepressants, LTP, LTD, dendritic spines, glutamate receptors

## Abstract

Antidepressant drugs are usually administered for several weeks for the treatment of major depressive disorder. However, they are also prescribed in several additional psychiatric conditions as well as during long-term maintenance treatments. Antidepressants induce adaptive changes in several forebrain structures which include modifications at glutamatergic synapses. We recently found that repetitive administration of the selective serotonin reuptake inhibitor (SSRI) fluoxetine to naïve adult male rats induced an increase of mature, mushroom-type dendritic spines in several forebrain regions. This was associated with an increase of GluA2-containing α-amino-3-hydroxy-5-methylisoxazole-4-propionate receptors (AMPA-Rs) in telencephalic postsynaptic densities. To unravel the functional significance of such a synaptic re-arrangement, we focused on glutamate neurotransmission in the hippocampus. We evaluated the effect of four weeks of 0.7 mg/kg fluoxetine on long-term potentiation (LTP) and long-term depression (LTD) in the CA1 hippocampal subfield. Recordings in hippocampal slices revealed profound deficits in LTP and LTD at Schaffer collateral-CA1 synapses associated to increased spine density and enhanced presence of mushroom-type spines, as revealed by Golgi staining. However, the same treatment had neither an effect on spine morphology, nor on LTP and LTD at perforant path-CA1 synapses. Cobalt staining and immunohistochemical experiments revealed decreased AMPA-R Ca^2+^ permeability in the *stratum radiatum (s.r.)* together with increased GluA2-containing Ca^2+^ impermeable AMPA-Rs. Therefore, 4 weeks of fluoxetine treatment promoted structural and functional adaptations in CA1 neurons in a pathway-specific manner that were selectively associated with impairment of activity-dependent plasticity at Schaffer collateral-CA1 synapses.

## Introduction

Fluoxetine is a selective serotonin reuptake inhibitor (SSRI) that is widely used to treat anxiety- and mood-related disorders, but in addition, its use has been expanded to other psychiatric conditions and is often continued after remission of symptoms (Schatzberg, 2000; Blier et al., 2007). The therapeutic effect of antidepressant drugs is mediated by cellular events which include adult hippocampal neurogenesis, maturation of nascent neurons and changes in gene expression. It has been shown that the activation of gene transcription following fluoxetine treatment is elicited by epigenetic remodeling of chromatin structure leading to increased plasticity and dendritic spine remodeling (Maya Vetencourt et al., 2011; Wang et al., 2011). This has been proposed to underlie plastic changes in glutamate neurotransmission (Pittenger and Duman, 2008; Sanacora et al., 2008; Maya-Vetencourt et al., 2012).

It had been previously described by us that 28 days of 0.7 mg/kg fluoxetine administration to adult naïve rats induced growth of dendritic spines and changes in glutamate receptor subunit composition in cerebrocortical synapses (Ampuero et al., 2010). In addition, such changes led to impairment of remote, but not recent, hippocampus-dependent memory (Ampuero et al., 2013). To study the effect of fluoxetine on cellular plasticity, we focused on CA1 pyramidal neurons which receive spatially segregated direct and indirect excitatory inputs from the entorhinal cortex (EC) *via* the perforant path and the Schaffer collaterals originating in CA3, respectively. While Schaffer collaterals terminate in the CA1 *stratum radiatum (s.r.)* on proximal dendrites, the direct perforant path makes synapses on the distal dendrites of CA1 neurons that are localized in the *stratum lacunosum moleculare (s.l.m.)* (Steward, 1976).

The induction of long-term potentiation (LTP) and long-term depression (LTD) of excitatory synaptic transmission at CA1 requires both N-methyl-D-aspartate receptor (NMDA-R) activation and trafficking of α-amino-3-hydroxy-5-methylisoxazole-4-propionate receptors (AMPA-Rs) (Citri and Malenka, 2008). NMDA-Rs are heterotetramers composed mandatorily of two GluN1 and two GluN2 subunit types (A to D), whereas AMPA-Rs are comprised of four unique subunits, GluA1-GluA4. In the adult hippocampus, AMPA-Rs are heterotetramers assembled preferentially by GluA1 and GluA2/GluA3 subunits. Subunit composition is a major determinant of biophysical channel properties, downstream signaling, receptor trafficking and synaptic plasticity. In such a way, GluA2-containing receptors are Ca^2+^ impermeable, in contrast to GluA2-lacking channels that are permeable to the divalent ion (Traynelis et al., 2010). High frequency synaptic stimulation induces LTP via NMDA-R-dependent Ca^2+^ influx, activation of several protein kinases and recruitment of GluA1-containing AMPA-Rs to the synapse. In contrast, LTD is induced by low frequency synaptic stimulation, prolonged increases in calcium levels leading to protein phosphatase activation and GluA1- and GluA2-containing AMPA-R internalization (Derkach et al., 2007; Isaac et al., 2007; Citri and Malenka, 2008). Although the contribution of specific AMPA-R subunits is not firmly established for LTD, GluA1 subunits at least play an important role (Meng et al., 2003; He et al., 2011). Additionally, LTP and LTD are accompanied by opposing changes in spine density and morphology, i.e., spine length, head volume, and neck diameter. As such, LTP correlates with growth of new spines and enlargement of preexisting spines whereas LTD associates with shrinkage and loss of spines (Segal, 2005).

Based on the fluoxetine-induced enrichment of GluN2A over GluN2B-containing NMDA-Rs and GluA2 over GluA1-containing AMPA-Rs (Ampuero et al., 2010) associated to increased mushroom-type spines, we hypothesized that such changes might negatively affect further potentiation. Therefore, we examined the consequences of repetitive fluoxetine treatment on spine morphology as well as LTP and LTD in the proximal and distal dendrites within the CA1 *s.r*. and *s.l.m*., respectively. Our data indicated that fluoxetine did not induce structural nor functional changes at perforant path-CA1 synapses. However, consistent with our hypothesis, increased mushroom-type dendritic spines on proximal dendrites, where Schaffer collateral to CA1 synapses occur in the *s.r*. were associated with a significant reduction of LTP. Interestingly, LTD was also impaired at these synapses. In addition, AMPA-R-dependent Ca^2+^ permeability was decreased specifically in the *s.r*. along with increased GluA2 content in AMPA-Rs.

## Materials and methods

### Animals

Adult male Sprague–Dawley rats weighting 280–300 g (~12 weeks old) at the beginning of the fluoxetine treatment were used for all experiments. All procedures involving animals were approved by the Universidad de los Andes Bioethical Committee and were performed in accordance with the National Institute of Health Guide for the Care and Use of Laboratory Animals (NIH Publications No. 80-23). Either saline (0.9% NaCl, control group) or 0.7 mg/kg fluoxetine (Ely-Lilly Co., Indianapolis, USA) dissolved in saline was administered daily via i.p. injection between 9:00 and 10:00 AM for 28 days (flx 4 weeks). A second control group of rats received a single fluoxetine injection (flx 24 h).

In total, 85 rats were sacrificed 24 h following the last fluoxetine or saline injection to perform electrophysiological recordings (*n* = 39), Golgi staining (*n* = 17), immunohistochemistry (*n* = 12), and cobalt staining (*n* = 17). For immunohistochemistry or for Golgi staining, rats were sacrificed under ketamine (50 mg/kg) and xylazine (10 mg/kg) anesthesia and then perfused intracardially with saline followed by 300 ml of 4% paraformaldehyde in PBS.

### Extracellular field recordings in the CA1 hippocampal subfield

Recordings were performed in hippocampal slices, as previously reported (Olmos et al., 2009). In each experimental group, 2–3 slices per rat were recorded. The animals were anesthetized with halothane gas and then intracardially perfused with artificial cerebrospinal fluid (aCSF, in mM; 124 NaCl; 5 KCl; 1.25 NaH_2_PO_4_; 1.0 MgCl_2_; 2.0 CaCl_2_; 26 NaHCO_3_; 10 glucose; pH 7.4). After perfusion, the animals were decapitated and brains rapidly removed, immersed in ice-cold dissection buffer (in mM; 212.7 sucrose; 5 KCl; 1.25 NaH_2_PO_4_; 3 MgSO_4_; 1 CaCl_2_; 26 NaHCO_3_; 10 glucose; pH 7.4) and then the hippocampi dissected to obtain transverse slices (400 μm thickness) with a vibratome (Campden Instruments, Leicester, UK). Slices were transferred to an interface chamber containing aCSF saturated with 95% O_2_/5% CO_2_ at 36°C, left at these conditions for 45 min, and then maintained at 24°C for 1 h. Single slices were transferred to a recording chamber containing aCSF and continually perfused at a flow of 2 ml/min.

Recording electrodes were glass micropipettes (1–3 MΩ) filled with aCSF. For CA3-CA1 experiments, the concentric bipolar stimulating electrode (FHC Corporate and Manufacturing, Bowdoin, ME, USA) was placed on the Schaffer collateral fibers, and the recording electrode into the *s.r*. of the CA1 region. For input-output (I/O) experiments, increasing current steps from 0 to 500 μA were applied. Averaged fiber volley amplitude vs. fEPSP slope was plotted. For LTP experiments, stimuli able to elicit 50% of the maximum fEPSP response were used for baseline recordings and theta burst stimulation (TBS). LTP was elicited after 20 min of a stable baseline using electrical stimulation (constant current, 200 μs stimuli) delivered every 15 s. The TBS protocol consisted of five stimuli trains with an inter-train interval of 10 s. Each train consisted of 10 bursts at 5 Hz, each burst having four pulses at 100 Hz. After TBS, potentiation was recorded for 1 h. For LTD experiments, the stimulus intensity was adjusted to elicit 80% of the maximum response for baseline recordings and low frequency stimulation (LFS). LTD was elicited after 20 min of a stable baseline using electrical stimulation (constant current, 200 μs stimuli) delivered every 30 s. The LFS protocol to induce LTD consisted of 1200 pulses at 2 Hz and thereafter, data acquisition lasted 60 min. To test the plasticity of the direct pathway from the EC to CA1 synapses, the stimulation electrode was placed on the temporoammonic pathway while recordings were performed in the *s.l.m*. of the CA1 hippocampal area using the same stimulation protocol as in Schaffer collateral-CA1 *s.r*. recordings (Remondes and Schuman, 2002), but in this case the aCSF contained 4.0 mM CaCl_2_. Hippocampal slices were mechanically de-afferented from Schaffer collaterals.

Voltage recordings were acquired using an extracellular amplifier (Dagan Corporation, Minneapolis, MI, USA) and a data acquisition board (National Instruments, USA) controlled through Igor Pro software (Wavemetrics Inc., USA). LTP and LTD plots were obtained by measuring the fEPSP slopes, considering the baseline as 100%.

### Spine density and morphology

Brains were processed using the FD Rapid GolgiStainTM kit (FD Neuro Technologies, Baltimore, USA) and analyzed as already described (Ampuero et al., 2007, 2010). Briefly, 11 selected pyramidal neurons of hippocampal layer CA1 were examined per experimental condition. Proximal secondary dendrites in the *s.r*. that emerged from the primary dendrite at <50 μm away from the soma were examined. Distal dendrites were at >150 μm away from the soma and corresponded to *s.l.m*. dendrites. It is known that histological techniques lead to shrinkage due to fixation, processing, dehydration, and mounting of samples. In our case, the dentate gyrus was clearly visible at about 380 μm away from the pyramidal cell layer, indicating that at stretches between 200 and 350 μm we are positioned in the *s.l.m*. In two cases, based on a second criterion used to select *s.l.m*. dendrites: i.e., that *s.r*. pyramidal cell dendrites run perpendicular to the hippocampal fissure while this perpendicular orientation is absent in *s.l.m*. (Pyapali et al., 1998; Megias et al., 2001), selected dendrite stretches began at ~160 μm away from the cell body. Spines were quantified along segments of 50 μm. Spine density was calculated as number of spines per μm and spine shape classification was performed under the microscope at different focal planes into three groups: (1) filopodia/thin, (2) stubby and (3) mushroom/branched. In a few cases (less than 10%) in which spine category was not clear to the observer, spine, and neck diameters were measured at the focal plane in which the image appeared at its maximal size and classified according to measures reported by Harris (Harris et al., 1992). Photomicrographs for representative images were captured with a ZeissAxioplan 2 microscope (100 × objective, numerical aperture 1.3) attached to a Nikon COOLPIX995 digital camera (final magnification of 4700×).

### Immunostaining

Anti-GluA1 (1:50) and anti-GluA2 (1:400) from Millipore (Temecula, CA, USA) for immunofluorescence and immunohistochemical staining were used. For immunofluorescence, the secondary antibody Alexa Fluor® 488 conjugated to goat anti-rabbit IgG (1:400) from Invitrogen (Eugene, OR, USA) was used. Fluorescent images were obtained on a confocal microscope (LSM 510 Meta, Zeiss, USA; 20 × magnification) using LSM 5 Pascal software. For immunohistochemistry, primary antibodies were detected with biotinylated anti-rabbit secondary antibody (1:200, Jackson ImmunoResearch Laboratories, Inc., West Grove, PA, USA) and 3,3-diaminobenzidine tetrahydrochloride (DAB) using a nickel-intensified reaction according to the published protocol (Ampuero et al., 2010). For quantification of immunochemical staining, brain slices were visualized under a light microscope (AxiosKop, Zeiss, Germany; 10× magnification; numeric aperture 0.3), and images of serial sections were captured with a digital camera (Nikon, CoolPIX 995) with a final magnification of 37×. Digitized images were analyzed with the NIH ImageJ software. Optical density of hippocampal *s.r*., *s.l.m*., and *stratum pyramidale (s.p.)* layers in CA1 was analyzed from 3 to 5 sections per animal restricted to bregma −3.14 mm and bregma −4.52 mm and five consecutive areas of 47.3± 1.6 μm^2^ were quantified. In the case of the *s.l.m*., these areas were selected at 30–80 μm away from the hippocampal fissure, perpendicular toward the *s.p*. cell layer.

### Cobalt staining

The Ca^2+^ permeability of GluA1-containing AMPARs was evaluated in fresh hippocampal sections using a modified cobalt staining technique, adapted from Osswald et al. (2007). Briefly, three to five slices per animal (*n* = 9 animals per condition) were incubated with 5 mM CoCl_2_ in assay buffer for 10 min in presence of a NMDA-R antagonist (40 μM aminophosphonovalerate, APV), a voltage-dependent Na^+^ channel blocker (1 μM tetrodotoxin), and a Ca^2+^ channel blocker (2 μM Nimodipine). The AMPA-R antagonist, 6-cyano-7-nitroquinoxaline-2,3-dione (20 μM CNQX), was added to negative controls. Sections were incubated with the AMPA-R agonist quiscualic acid (100 μM) for 20 min. Intracellular Co^2+^ was precipitated with 0.24% ammonium sulfide, and slices were fixed in 0.8% glutaraldehyde. Silver enhancement of the Co^2+^ sulfide precipitate was obtained with AgNO_3_. Photomicrographs of hippocampal sections were captured with a stereoscopic microscope attached to a Nikon COOLPIX995 digital camera at a final magnification of 3.74×. For each hippocampal digital image, the dorsal-most CA1 subfield was selected and the mean optical density of 114.0 × 28.5 μm (3250 μm^2^) rectangles was calculated for each layer, i.e., *s.p*., *s.l.m., and s.r*., and using NIH ImageJ software.

### Statistical analysis

The GraphPad PRISM 4.0 software was used to analyze data. Data are presented as mean ± SEM. For morphological and optical density analysis, the Mann-Whitney *U*-test was applied. In the electrophysiological experiments *n-value* represents the mean of 2–3 slices per rat per condition. For input/output curves, data from each experimental group was fit to a linear regression and compared using covariate analysis. LTP and LTD data was subjected to one-way analysis of variance (ANOVA), followed by a Bonferroni *post-hoc* test.

## Results

### Fluoxetine induced changes in spine density and mushroom-type morphology in the CA1 *s.r.*, but not in the *s.l.m.*

**Figure 1:**
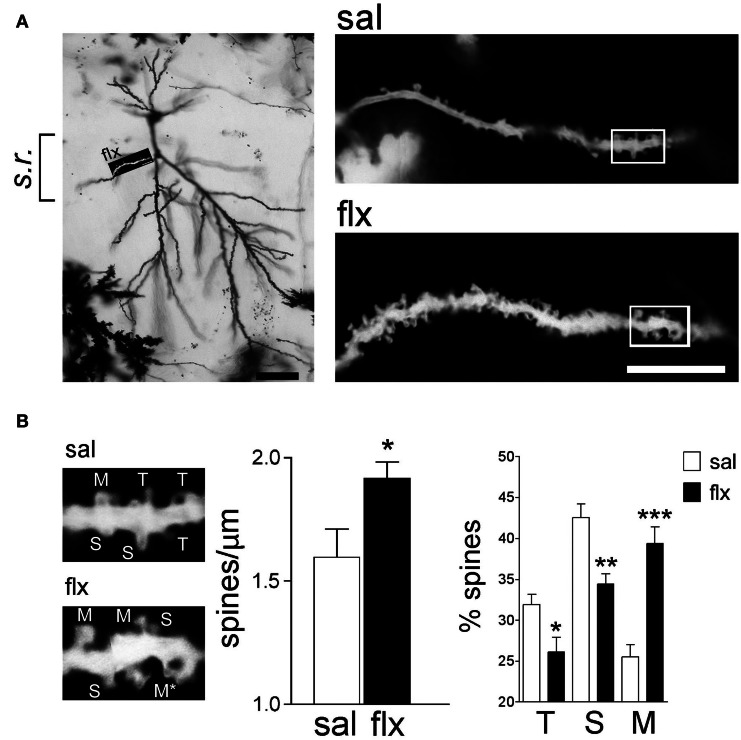
**Long-term fluoxetine treatment induces morphological changes in proximal dendritic spines of CA1 pyramidal neurons. (A)** Left: A representative image of a Golgi stained neuron from a fluoxetine-treated rat. CA1 s*tratum radiatum* (*s.r*.) subfield was chosen for analysis. Scale bar: 50 μm. Right: representative dendritic segments of saline and fluoxetine-treated rats under larger amplification. Scale bar: 10 μm. **(B)** The selected dendritic segments in **(A)** (right panels) are shown with identified spine types: filopodia/thin (T), stubby (S), and mushroom/branched (M). The asterisks indicate two superimposed M-type spines that were resolved by observation at different focal planes. Bar graphs show spine density and the abundance of spines in each of the three shape categories. Results are presented as mean ± SEM and were determined from 11 cells per condition, obtained from eight saline (total spines, 874)- and nine fluoxetine (total spines, 957)-treated rats. Data were statistically evaluated with the Mann–Whitney *U*-test, ^*^p < 0.05, ^**^p < 0.01, ^***^p < 0.001.

**Figure 2:**
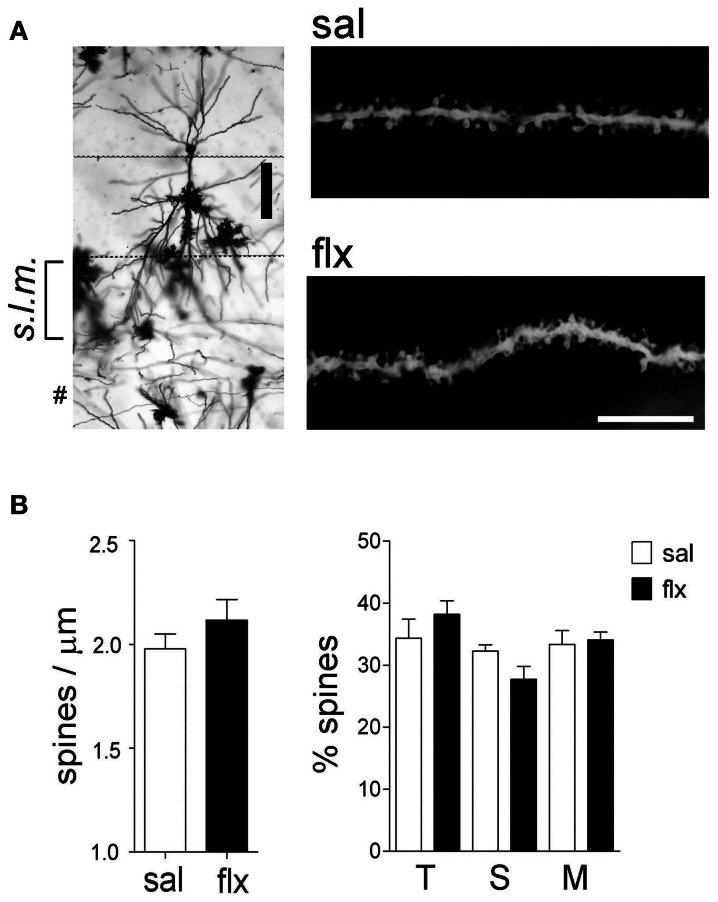
**Long-term fluoxetine treatment does not affect distal dendritic spine morphology of CA1 pyramidal neurons. (A)** Left: Golgi stained CA1 pyramidal neuron indicating the *stratum lacunosum moleculare* (*s.l.m.)* where the morphological analysis was performed. ^#^At this level can be observed granule neuron dendrites coming from dentate gyrus. Scale bar: 100 μm. Right: representative dendritic segments of saline and fluoxetine-treated rats under larger amplification. Scale bar: 10 μm. **(B)** The number of spines and the morphology of spines were analyzed and plotted as spine density and % of spine type, respectively. Filopodia/thin (T), stubby (S), and mushroom/branched (M) shape categories are shown. Results are presented as mean ± SEM and were determined from 10 to 11 cells per condition, obtained from five saline (total spines, 979)- and six fluoxetine (total spines, 942)-treated rats. Data were not statistically significant after a Mann–Whitney *U*-test analysis.

In order to examine the morphology of the post-synaptic compartment in CA1, i.e., dendritic spines, the Golgi staining method was used. Spine morphology and spine density of secondary dendrites was analyzed at proximal segments emerging <50 μm away from the soma of hippocampal CA1 neurons (Figure 1A). Spine density (spines/μm) increased significantly from 1.59 ± 0.11 to 1.92 ± 0.07 after 4 weeks of fluoxetine treatment (*p* < 0.05). This increase was accompanied by a higher proportion of mushroom-type spines (saline: 26.2 ± 1.7%; fluoxetine: 39.6 ± 1.8%, *p* < 0.001) and a concomitant decrease of thin (saline: 30.7 ± 1.4%; fluoxetine: 26.1 ± 1.4%; *p* < 0.05) and stubby spines (saline: 43.1 ± 2.3%; fluoxetine: 34.3 ± 1.1%; *p* < 0.01) (Figure 1B). These observations indicated that repetitive fluoxetine administration induced synaptogenesis and synapse maturation in the hippocampal CA1 *s.r*., similarly to those reported in some cerebrocortical regions (Ampuero et al., 2010). To test whether changes in spine morphology extended to distal dendrites, spine density and shape were quantified on secondary dendrites emerging at distances farther than 150 μm away from the cell soma, located in the *s.l.m*. (Figure 2A). In contrast to the previous findings, no change in spine density nor in the proportion of morphological sub-types were found within the CA1 *s.l.m*. subfield (Figure 2B).

### Long-term fluoxetine treatment increased excitability at schaffer collateral-CA1 synapses, but impaired LTP and LTD

**Figure 3:**
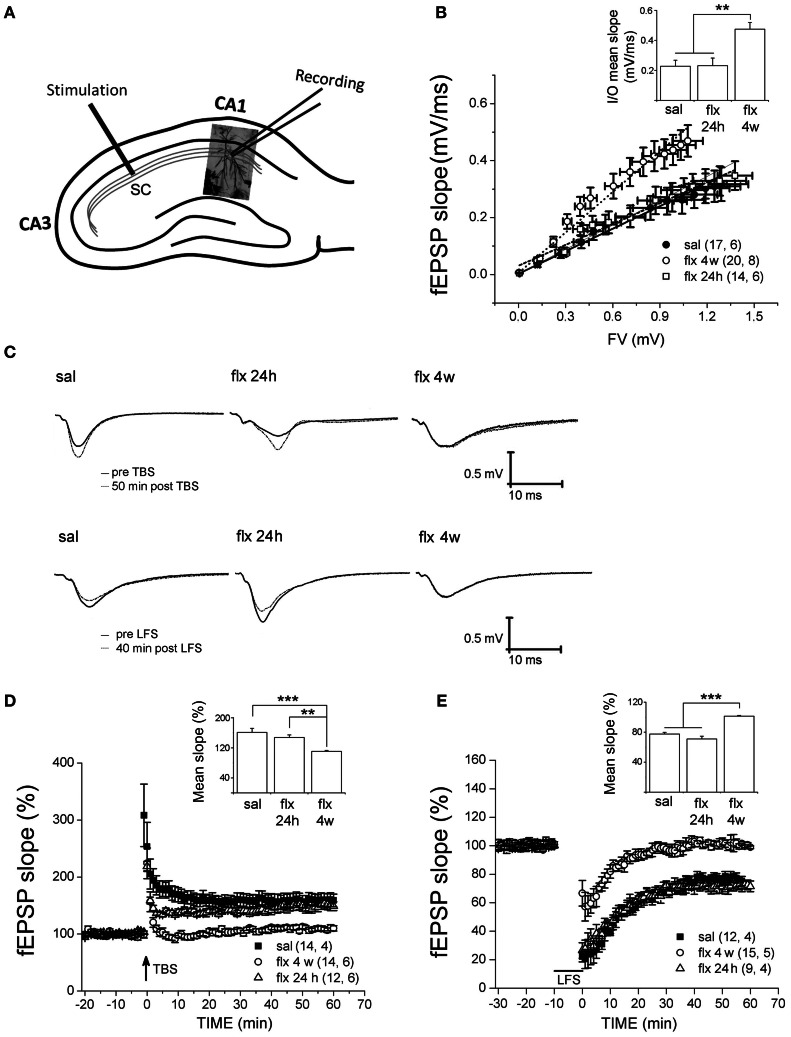
**Four weeks of fluoxetine administration affects basal synaptic transmission and synaptic plasticity at CA3-CA1 synapses.** Experiments were performed in slices from saline (sal), single (flx 24 h), and repeated (flx 4 weeks) fluoxetine treatment groups. **(A)** The diagram shows the stimulating electrode inserted into Schaffer collaterals (SC) and the recording electrode placed in the CA1 *s.r*. **(B)** Input/output curves. The fEPSP slopes vs. increasing presynaptic fiber volley (FV) amplitudes were plotted. Data were fit to a linear regression and compared using covariate analysis. The slope obtained in repetitive fluoxetine-treated rats (0.48 ± 0.05, *r*^2^ = 0.99) was significantly different (*p* < 0.01) compared to saline-treated rats (0.23 ± 0.04, *r*^2^ = 0.99) or to rats treated with fluoxetine only once (0.23 ± 0.05, *r*^2^ = 0.97). Saline, *n* = 6 (17 slices); fluoxetine 24 h, *n* = 6 (14 slices); fluoxetine 4 weeks, *n* = 8 (20 slices). **(C)** Representative traces of extracellular field recordings are shown before and after LTP (upper panels) or LTD (lower panels) induction. **(D)** Long-term potentiation (LTP) induced by theta burst stimulation (TBS) and **(E)** long-term depression (LTD) induced by low frequency stimulation (LFS) were blocked in hippocampal slices of rats treated for 4 weeks with fluoxetine. Bar graphs represent the mean % change in fEPSP slopes compared to baseline, from 45 to 60 min after LTP (**D** inset) or LTD induction (**E** inset). LTP: sal, *n* = 4 (14 slices); flx 4 weeks, *n* = 6 (14 slices); flx 24 h, *n* = 6 (12 slices). LTD: sal, *n* = 4 (12 slices); flx 4 weeks, *n* = 5 (15 slices); flx 24 h, *n* = 4 (9 slices). Data from **D** and **E** were statistically evaluated by one-way ANOVA, followed by a Bonferroni post hoc test. ^**^p < 0.01, ^***^p < 0.001.

To compare the effect of repetitive fluoxetine on basal neurotransmission and activity-dependent synaptic plasticity at CA3-CA1 synapses, we compared treated animals to two control groups: saline-treated rats and rats exposed to a single fluoxetine injection. Therefore, a direct effect of fluoxetine could be discarded. Stimulus-evoked fEPSPs were recorded at the CA1 *s.r.* after Schaffer collateral stimulation (Figure 3A). First, post-synaptic excitability was tested by I/O curves, in which the fEPSP slopes were measured in response to single electrical stimuli of increasing magnitude. Significantly higher fEPSP slopes were recorded after 28 days of fluoxetine treatment, as compared to both control groups (Figure 3B).

Application of the TBS protocol, which is capable of inducing robust LTP in saline-treated animals, was not able to induce LTP nor LTD in rats chronically treated with fluoxetine. In Figure 3C, representative recordings are shown. The mean % of fEPSP slopes, ranging from 45 to 60 min after the TBS, were pooled for analysis (saline: 161.3 ± 10.8%; fluoxetine 24 h: 147.7 ± 6.7%; fluoxetine 4 weeks: 110.7 ± 2.4%; *F*_(2, 14)_ = 16.04, *p* < 0.001; Figure 3D). A single fluoxetine injection did not interfere with LTP induction. A similar analysis of the LTD data revealed that LTD was blocked after repetitive fluoxetine treatment, but not after a single fluoxetine dose (saline: 77.5 ± 2.4%; fluoxetine 24 h: 71.0 ± 3.7%; fluoxetine 4 weeks: 101.3 ± 1.0%, *F*_(2, 11)_ = 46.23, *p* < 0.0001; Figure 3E). These results revealed that long-term fluoxetine, able to induce morphological changes in the post-synaptic compartment, induced deficits in activity-dependent plasticity. In turn, the increased excitability observed at CA3-CA1 synapses can be due to increased spine density and/or the fact that large spines are known to generate a larger postsynaptic responses (Spruston, 2008).

To further clarify whether the detrimental effects of fluoxetine on LTP and LTD were associated with the changes in spine morphology and density, electrophysiological recordings at perforant path-CA1 synapses were performed.

### Long-term fluoxetine treatment did not affect LTP nor LTD at perforant path-CA1 synapses

**Figure 4:**
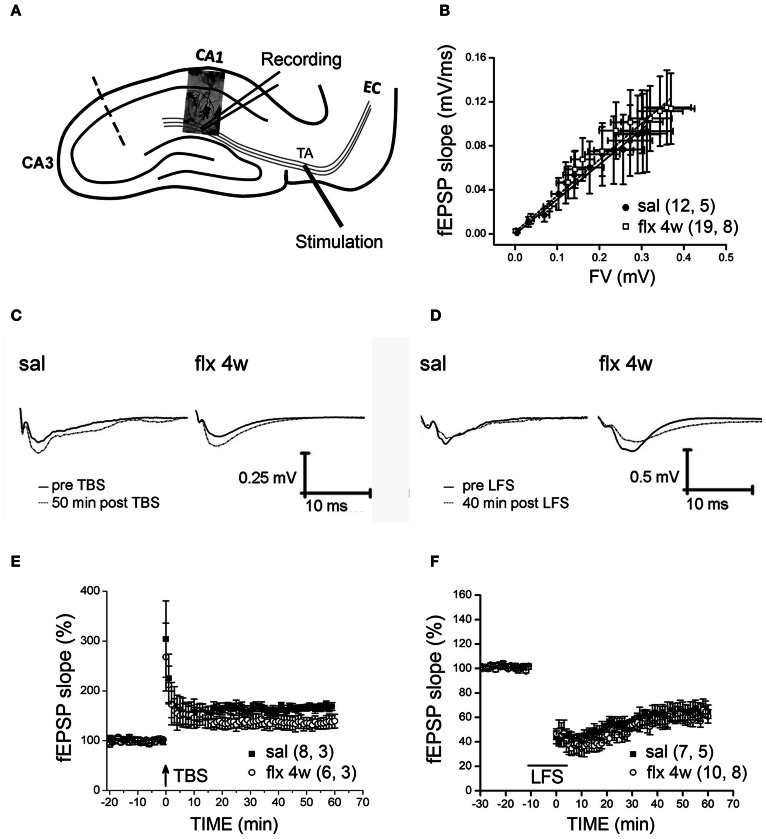
**Four weeks of fluoxetine administration does not affect synaptic plasticity at perforant pathway-CA1 synapses.** Experiments were performed in slices from saline- (sal) and fluoxetine- (flx 4 weeks) treated rats. **(A)** The diagram shows the stimulating electrode inserted into the temporoammonic pathway (TA) and the recording electrode in the CA1 *s.l.m*. EC, entorhinal cortex. The dashed line indicates the place of the physical de-afferentation of Schaffer collateral inputs to CA1. **(B)** The fEPSP slopes vs. increasing presynaptic fiber volley (FV) amplitudes were plotted to analyze the input/output curves. No significant difference between saline- and repetitive fluoxetine-treated rats was found after data were fit to a linear regression and compared using covariate analysis. Saline, *n* = 5 (12 slices); fluoxetine 4 weeks, *n* = 8 (19 slices). **(C,D)** Sample traces of extracellular field recordings are shown before and after LTP **(E)** or LTD **(F)** induction. **(E)** Long-term potentiation (LTP) induced by theta burst stimulation (TBS) were unaffected after perforant pathway stimulation. No significant difference was found when the mean fEPSP integrated between 45 and 60 min after LTP induction was compared (*p* = 0.143; unpaired *t*-test). LTP: sal, *n* = 3 (8 slices); flx 4 weeks, *n* = 3 (6 slices). **(F)** Similar LTD was also induced by low frequency stimulation (LFS) of the perforant path in slices from saline- and fluoxetine-treated animals (*p* = 0.519; unpaired *t*-test).

Stimulus-evoked fEPSPs were recorded at the CA1 *s.l.m.* after temporoammonic pathway stimulation (Figure 4A). No significant differences in fEPSP slopes were observed between saline- and fluoxetine-treated rats after 28 days of administration (saline: 0.3773 ± 0.1753; fluoxetine 4 weeks: 0.3838 ± 0.1335, *p* = 0.997; Figure 4B). LTP or LTD were induced in these synapses after TBS or LFS stimulation, respectively, in slices from saline- and repetitively fluoxetine-treated rats. In Figures 4C and D, representative recordings are shown. The mean % of fEPSP slopes, ranging from 45 to 60 min, indicated that both groups had similar LTP and LTD responses (LTP, saline: 166 ± 8.2%; fluoxetine 4 weeks: 136 ± 11.6%, *p* = 0.143; LTD, saline: 64.5 ± 6.8%; fluoxetine 4 weeks: 59 ± 4.6%, *p* = 0.519; Figures 4E and F). As mushroom-type spines are enriched in Ca^2+^ impermeable GluA2-containing AMPA-Rs and as such, should contribute to limited plasticity observed in the CA1 *s.r*., where changes in spine morphology had been detected along with LTP and LTD impairment (Isaac et al., 2007; Medvedev et al., 2008).

### Long-term fluoxetine treatment increased GluA2-containing AMPA-Rs in the CA1 *s.r.* subfield

**Figure 5:**
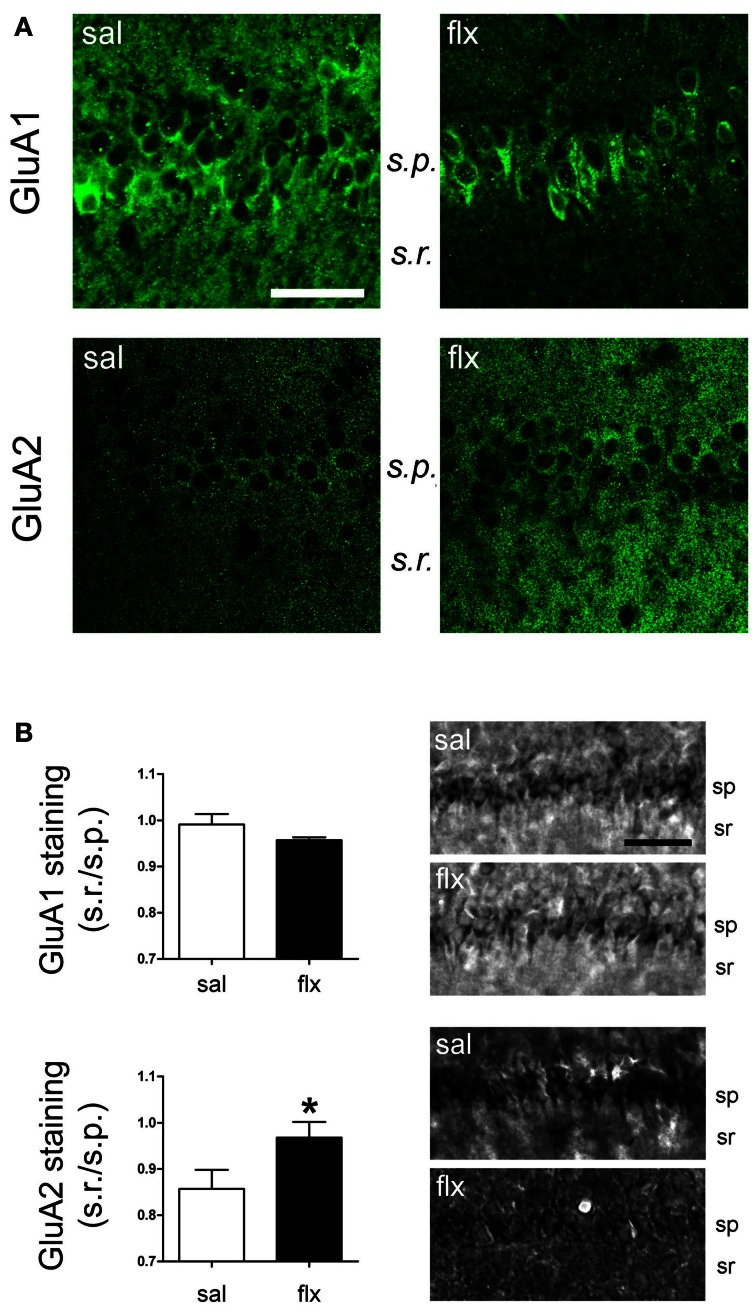
**GluA1 and GluA2 were detected in the CA1 region by immunofluorescence and immunohistochemical staining. (A)** Fluorescence images of GluA1 and GluA2 immunostainings reveal a change in the distribution of subunits between the cell soma (*s.p*.) and dendritic (*s.r*.) compartments. **(B)** Relative staining intensity (*s.r*./*s.p*.) of immunohistochemical GluA1 and GluA2 stainings revealed higher GluA2 intensity in the *s.r*. of fluoxetine-treated rats (^*^p < 0.05, *n* = 5−7, Student *t*-test). *s.r*., *stratum radiatum*; *s.p., stratum pyramidale*. Scale bars: 50 μm.

**Figure 6:**
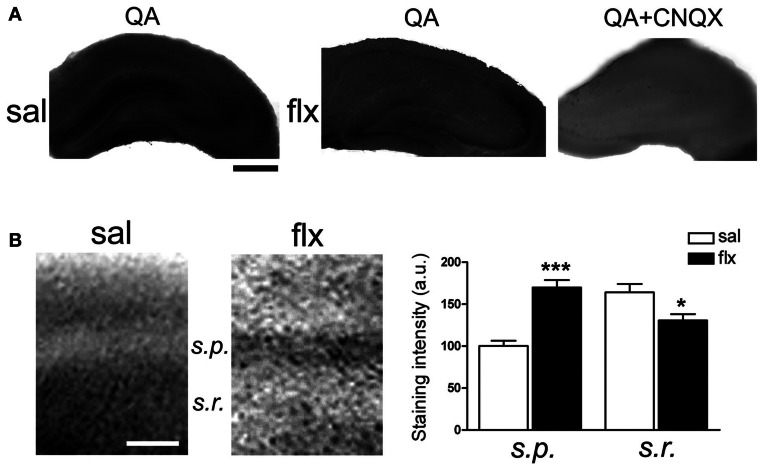
**Long-term fluoxetine administration decreased Ca^2+^-permeable AMPA-Rs in the CA1 *stratum radiatum (s.r.)*.** Hippocampal slices from saline- (sal) and fluoxetine- (flx) treated rats were stimulated with 100 μM quisqualic acid (QA) to detect Co^2+^ uptake. The AMPA-R blocker CNQX (20 μM) was used as a negative control (QA+CNQX). **(A)** Hippocampal sections are shown in each experimental condition. Bar: 500 μm. **(B)** Increased magnification was used to quantify relative staining in the two CA1 subfields: *stratum pyramidale (s.p.)* and *s.r*. Bar: 50 μm. Bar graphs: the mean optical density was plotted. Data were statistically evaluated with the Mann–Whitney *U*-test, ^*^p < 0.05, ^***^p < 0.001. (*n* = 9 group).

Immunofluorescent labeling of GluA1 and GluA2 subunits was performed in brain sections after 4 weeks of either saline or fluoxetine administration. Confocal images revealed decreased GluA1, but enhanced GluA2 levels in the CA1 *s.r*. subfield following fluoxetine treatment (Figure 5A). To quantify the relative content of these subunits in hippocampal subfields, immunohistochemical staining was performed. The relative staining intensity compared to the *s.p*. in the *s.r*. and in the *s.l.m*. was assessed. While GluA1 staining did not change in neither of the subfields, increased GluA2 immunoreactivity was found in the *s.r*. of fluoxetine-treated rats (Figure 5B, 0.82 ± 0.04 vs. 1.01 ± 0.05; *p* < 0.05). In contrast, no staining difference was found in the *s.l.m*. (not shown, 1.13 ± 0.1 vs. 1.14 ± 0.08, *n* = 5). Interestingly, as already reported, these data revealed an increased AMPA-R subunit staining in the control situation in the *s.l.m*. when compared to the *s.r*. (0.82 ± 0.04 vs. 1.13 ± 0.1; *p* < 0.05) (Nicholson et al., 2006). To further explore a higher GluA2 over GluA1 content at the functional level, the AMPA-R Ca^2+^-permeability was assessed by using the cobalt staining protocol (Figure 6). AMPA-Rs were stimulated with quisqualic acid while other Ca^2+^ influx pathways were pharmacologically blocked. After repetitive fluoxetine treatment, higher cobalt staining in the CA1 *s.p*. (saline: 100.3 ± 6.2%, fluoxetine: 169.9 ± 8.9%; *p* < 0.001) was found while the staining intensity in the CA1 *s.r*. (saline: 164.0 ± 10.1%, fluoxetine: 130.6 ± 7.5%; *p* < 0.05) decreased. No differences were found among experimental groups in the *s.l.m*. (not shown, 168 ± 16% vs. 163 ± 17%). All together, these results indicate that GluA2 is up-regulated in the *s.r*. while no staining differences could be detected in the *s.l.m*.

## Discussion

The main finding of the present work is that repetitive, but not a single, administration of low and clinically relevant doses of fluoxetine to naïve rats enhanced the excitability of CA1 neurons, but impaired activity-dependent plasticity at hippocampal CA1 synapses in an input-specific manner. This impairment was associated to a selective increase of spine density and to a higher proportion of mushroom-type spines, restricted to the proximal dendritic segment of CA1 pyramidal cells that receives the Schaffer collateral input. At the molecular level, changes in AMPA-R stoichiometry in the *s.r*. might contribute to the described effect. An increased GluA2 over GluA1 content in AMPA-Rs, associated with enhanced dendritic spine density was reported previously in the telencephalon by western blots of immunoprecipitated receptor subunits from postsynaptic densities and in isolated synaptosomes. In turn, immunohistochemistry and morphological analysis revealed that changes were restricted to some neocortical regions, while the hippocampus, with its well-defined cellular layers and dendritic sub-regions, was not analyzed (Ampuero et al., 2010). To now explore a functional implication of such molecular and morphological adaptations, we took advantage of the well-characterized activity-dependent cellular plasticity paradigms in the hippocampal CA1 region.

### Homeostatic synaptic adaptations at the CA1 hippocampal subfield are possibly induced by long-term fluoxetine treatment

The CA1 hippocampal region is densely innervated by serotoninergic fibers originating in the dorsal raphe nuclei (Jacobs and Azmitia, 1992), which regulate excitability and synaptic transmission via activation of a large range of receptor subtypes. An enhanced serotonin-mediated synaptic response is caused by 5-HT7 and 5-HT4 receptors (Torres et al., 1995; Costa et al., 2011), while 5-HT1A and 5-HT1B activation results in hyperpolarization and, hence, decreased excitability (Andrade and Chaput, 1991; Mlinar et al., 2003). Interestingly, the integration of excitatory and inhibitory serotonin inputs resulted in global inhibition of CA1 neurons that could be observed either following activation of the raphe nuclei *in vivo* (Segal, 1975) or after local serotonin application (Otmakhova and Lisman, 2000; Otmakhova et al., 2005), supporting the idea of a homeostatic up-scaling induced by long-term elevation of serotonin levels (Turrigiano et al., 1998). Therefore, serotonin-mediated tonic inhibition of glutamatergic CA1 pyramidal neurons could trigger an adaptive homeostatic response leading to enhanced basal neurotransmission and dendritic spine remodeling (Turrigiano, 1999). However, how this remodeling can be performed in a differential manner along the dendritic tree is presently unknown. A differential serotoninergic innervation of *s.l.m*. dendrites compared to the *s.r*. dendrites as well as possible serotonin receptor subtype gradients might contribute to such effects (Moore and Halaris, 1975; Oleskevich and Descarries, 1990). Gradients of intrinsic membrane conductances and of glutamate receptors along the dendritic tree in the CA1 region will also influence post-synaptic responses and activity-dependent plasticity (Nolan et al., 2004; Nicholson et al., 2006; Camp, 2012). In addition, the mechanisms that contribute to plasticity are differentially expressed along both afferent pathways that contact CA1 apical dendrites: while an enhancement of pre-synaptic glutamate release contributes to perforant path-CA1 LTP, this does not occur at CA3-CA1 LTP (Ahmed and Siegelbaum, 2009), although both are NMDA-R dependent. A similar effect of fluoxetine on spine morphology in the CA1 *s.r*. had already been described (Hajszan et al., 2005), however, the CA1 *s.l.m*. was not analyzed nor input-specific electrophysiological recordings were performed. The fact that LTP was unaffected in the direct perforant pathway input to the CA1 region, at least until 60 min after induction, favors the idea that the deficit in synaptic plasticity at CA3-CA1 synapses was associated to changes in postsynaptic spine morphology, i.e., that stronger and more “mature” synapses negatively affected further activity-dependent plasticity at CA3-CA1 synapses.

### Participation of AMPA-Rs in fluoxetine-induced effects

In agreement with a homeostatic-like synaptic re-organization induced by inhibitory-acting serotonin, synaptic scaling following the blockade of network activity is mediated by an accumulation of GluA2-containing AMPA-Rs (Gainey et al., 2009). In such a way, Ca^2+^ entry is abolished in AMPA-Rs containing solely GluA2 (Traynelis et al., 2010). Moreover, GluA2 deficient mice exhibited increased Ca^2+^ permeability and enhanced LTP (Jia et al., 1996). Therefore, the increase of GluA2 staining in dendrites is consistent with the data obtained with cobalt measurements assessing Ca^2+^ permeability. It had been shown that repetitive fluoxetine treatment increased the expression of AMPA-R subunits in the forebrain, including the prefrontal cortex and hippocampus (Ampuero et al., 2010; Barbon et al., 2011). However, the fact that GluA2 staining and Ca^2+^ permeability was increased and decreased, respectively, in the *s.r*. is in line with the change in fluoxetine-induced AMPA-R subunit composition reported to occur in isolated synaptic fractions derived from the whole forebrain (Ampuero et al., 2010). Thus, reduced AMPA-R dependent Ca^2+^ influx in CA1 dendrites could contribute to the impairment in activity-dependent plasticity. The participation of other Ca^2+^-permeable ion channels, e.g., NMDA-Rs and voltage-sensitive Ca^2+^-channels, cannot be excluded. Interestingly, it has been shown that the latter can regulate Ca^2+^ influx into dendritic spines in a homeostatic manner (Yasuda et al., 2003). Moreover, along with a change in AMPA-R subunit composition, fluoxetine also induces a switch toward GluN2A-rich NMDA-Rs (Ampuero et al., 2010), which are characterized by a reduced Ca^2+^ influx when compared to GluN2B subunit-containing receptors.

The prevailing view regarding the participation of AMPA-R subunits in LTP and LTD establishes that GluA1 subunits become inserted or endocytosed to the synapse in an activity dependent manner while GluA2/GluA3 subunits traffic constitutively. Double GluA2/3 knockout animals continue to express LTP and LTD (Meng et al., 2003), while GluA1 availability and trafficking is required for early expression of LTP and LTD (Derkach et al., 2007). Recent findings indicate that LTP depends more importantly on the presence of an AMPA-R reserve pool than on specific subunits (Granger et al., 2013). Taken together, the effect of fluoxetine could be due to a deficiency in GluA1 subunit trafficking and/or membrane insertion leading to a relative lack of extra-synaptic AMPA-Rs.

### Effect of fluoxetine on plasticity paradigms

Our study revealed that 28 days of fluoxetine impaired activity-dependent plasticity that was associated with changes in spine morphology. This is in opposition to two previous studies which reported that fluoxetine restored developmental- or juvenile-like plasticity in the visual cortex or lateral amygdala, respectively, in adult rodents (Maya Vetencourt et al., 2008; Karpova et al., 2011). In the first study, Maya Vetencourt et al. used a similar treatment duration and doses, although the rat strain was different (Maya Vetencourt et al., 2008). In the second study, Karpova et al. administered fluoxetine to juvenile mice (P42) at a 14-fold higher dose for 3 weeks (Karpova et al., 2011). Therefore, differences in the age of animals, treatment duration, examined brain area, as well in the strains of rats or rodent species used may critically influence the outcomes of the experiments. Especially differences in the density of serotoninergic innervation and in the expression of serotonin receptor subtypes might define the consequences of fluoxetine-induced serotonin elevation. Indeed, the apparent contradictory effect of repetitive fluoxetine on LTP (i.e., facilitator in the lateral amygdala but blocker in the CA *s.r*.) might be explained by the differential effect of serotonin in these regions. While serotonin activated excitatory principal neurons of the lateral amygdala by enhancing depolarization through 5-HT_2_ receptors (Yamamoto et al., 2012), it activated inhibitory interneurons in the hippocampal CA1 subfield (Shen and Andrade, 1998) and acted on inhibitory receptors expressed by CA1 pyramidal neurons. In the visual cortex, reduced intra-cortical inhibition was associated with the effect of chronic fluoxetine treatment on restoration of LTP in the visual cortex (Maya Vetencourt et al., 2008). The role of the inhibitory tone at CA3-CA1 synapses was not tested in the present study.

Another study showed that repeated fluoxetine administration using a dose of 1 mg/kg, close to the dose used by us, reduced LTP at perforant path-dentate gyrus synapses (Stewart and Reid, 2000). The idea that in the CA1 region serotonin inhibited LTP was discussed in a previous *in vivo* study, in which acute fluoxetine administration (10 mg/kg, i.p., 40 min prior to Schaffer collateral stimulation) increased serotonin levels, which, in turn, blocked CA1 LTP (Shakesby et al., 2002). This blocking effect could be ascribed to immediate elevation of serotonin, while a single fluoxetine administration, even at higher doses, should not lead to maintained high serotonin levels 24 h after the injection (Caccia et al., 1990). This suggested that the effect we observed after 4 weeks of administration, but measured 24 after the last dose, was a consequence of adaptive plastic changes that required repeated fluoxetine administration, and not to acute changes in serotonin. Consistent with our data, it was shown that repeated administration of methamphetamine, a drug that acts on central monoamine neurotransmission, including the serotonin system, enhanced basal neurotransmission and had a detrimental effect on CA3-CA1 synaptic plasticity (Swant et al., 2010).

The increased fEPSPs revealed in I/O curves in fluoxetine-treated animals could be due to direct effects of fluoxetine on glutamate receptors. However, fluoxetine has inhibitory effects on AMPA and NMDA receptors (Szasz et al., 2007; Kim et al., 2013) and therefore, decreased fEPSPs should be expected. In consequence, the most straightforward explanation is that larger spines are able to elicit larger post-synaptic responses. This could be mediated by several plasticity-related signals that are expressed following fluoxetine treatment, such as BDNF (Castren, 2005; Bath et al., 2012) or IGF-1 (Corvin et al., 2012), which in turn are able to stimulate glutamate receptor expression and receptor phosphorylation (Slack et al., 2004; First et al., 2011). A changed inhibition due to serotonin-induced re-arrangements of interneurons can also contribute to this phenomenon (Mendez et al., 2012).

The concept of meta-plasticity posits that synapses that have previously been potentiated are more likely to express LTD and less likely to express LTP, whereas the opposite applies if LTD has been previously induced (Bear, 2003). It is therefore difficult to reconcile that both plasticity paradigms are impaired under the used experimental conditions. If LTP is occluded due to a synaptic up-scaling at CA3-CA1 synapses, LTD should be enhanced, but not decreased, at these synapses. We therefore propose that Ca^2+^-influx pathways and/or Ca^2+^ changes in dendritic spines caused by intracellular release or changes in buffering capacity, might be altered thereby affecting both plasticity paradigms. Further experiments, using different stimulation protocols, increasing extracellular Ca^2+^ concentration or blocking inhibitory interneurons need to be performed to get insight into underlying mechanisms. However, it is clear that maintaining the stimulation conditions of the control group in fluoxetine-treated animals, both LTP and LTD are impaired. Similarly, a specific stimulation protocol and learning procedures can lead to occlusion of both LTP and LTD (Liang et al., 2002; Delvendahl et al., 2010), as we found in fluoxetine-treated animals.

In agreement with our findings, chronic escitalopram treatment inhibited CA3-CA1 LTP in healthy rats in a stress-resistant strain (Flinders Resistant Line rats, Karolinska Institutet). This phenomenon was attenuated after exposure to stress (Ryan et al., 2009), thereby confirming a distinct result in naïve subjects. Although the effect of fluoxetine described by us may be restricted to non-stressed or non-depressed subjects, we report that repetitive treatment impacts significantly glutamate neurotransmission and plasticity in the hippocampus.

### Conflict of interest statement

The authors declare that the research was conducted in the absence of any commercial or financial relationships that could be construed as a potential conflict of interest.
